# Attenuated Fatigue in Slow Twitch Skeletal Muscle during Isotonic Exercise in Rats with Chronic Heart Failure

**DOI:** 10.1371/journal.pone.0022695

**Published:** 2011-07-25

**Authors:** Morten Munkvik, Per Kristian Lunde, Jan Magnus Aronsen, Jon Arne Kro Birkeland, Ivar Sjaastad, Ole M. Sejersted

**Affiliations:** 1 Institute for Experimental Medical Research, Oslo University Hospital, Ullevål, Oslo, Norway; 2 Center for Heart Failure Research, University of Oslo, Oslo, Norway; 3 Department of Cardiology, Oslo University Hospital, Ullevål, Oslo, Norway; Universidad Europea de Madrid, Spain

## Abstract

During isometric contractions, slow twitch soleus muscles (SOL) from rats with chronic heart failure (chf) are more fatigable than those of sham animals. However, a muscle normally shortens during activity and fatigue development is highly task dependent. Therefore, we examined the development of skeletal muscle fatigue during shortening (isotonic) contractions in chf and sham-operated rats. Six weeks following coronary artery ligation, infarcted animals were classified as failing (chf) if left ventricle end diastolic pressure was >15mmHg. During isoflurane anaesthesia, SOL with intact blood supply was stimulated (1s on 1s off) at 30Hz for 15 min and allowed to shorten isotonically against a constant afterload. Muscle temperature was maintained at 37°C. In resting muscle, maximum isometric force (F_max_) and the concentrations of ATP and CrP were not different in the two groups. During stimulation, F_max_ and the concentrations declined in parallel sham and chf. Fatigue, which was evident as reduced shortening during stimulation, was also not different in the two groups. The isometric force decline was fitted to a bi-exponential decay equation. Both time constants increased transiently and returned to initial values after approximately 200 s of the fatigue protocol. This resulted in a transient rise in baseline tension between stimulations, although this effect which was less prominent in chf than sham. Myosin light chain 2s phosphorylation declined in both groups after 100 s of isotonic contractions, and remained at this level throughout 15 min of stimulation. In spite of higher energy demand during isotonic than isometric contractions, both shortening capacity and rate of isometric force decline were as well or better preserved in fatigued SOL from chf rats than in sham. This observation is in striking contrast to previous reports which have employed isometric contractions to induce fatigue.

## Introduction

Limited exercise capacity is a hallmark of chronic heart failure (chf). Central hemodynamic deterioration with low cardiac output, reduced perfusion of working skeletal muscle and low anaerobic threshold likely contribute to the fatigue development in these patients. However, there is a poor relationship between clinical symptoms and left ventricular function, and ejection fraction shows only a weak correlation to maximal oxygen uptake [1], [2]. It has become clear that the muscle itself demonstrates pathological features, independent of cardiac function [3]. During heart failure skeletal muscle function is found to be altered both in experimental models and in patients. One main experimental finding is reduced exercise tolerance to isometric contractions [4]. Increased fatigue in this setting seems not to be due to reduced muscle perfusion [5].

Importantly, the energy demand of the muscle during isometric contractions is small compared to the energy demand during shortening (isotonic) contractions [6]. In addition there are reports that aerobic energy metabolism and mitochondrial function in skeletal muscle are less well tuned to meet the requirement for ATP during exercise in the heart failure condition [7]. One would therefore expect that muscle performance in the heart failure condition would be more impaired during shortening contractions both compared to normal muscle and compared to isometric contractions.

Since skeletal muscle fatigue is highly task dependent, functional impairment in normal muscle might also be underestimated if maximum force is the only fatigue output measured [8], [9]. In line with this, some argue that fatigue will develop more rapidly when it is induced by shortening contractions [10], [11]. Furthermore, alterations in shortening properties can develop independently of changes in isometric tension [12]–[14]. For these reasons we designed an experimental protocol where muscles can shorten upon stimulation with maintained perfusion and physiological temperature, allowing assessment of muscle shortening capacity (S_max_) as well as traditional fatigue parameters such as maximum isometric force (F_max_) [14].

The mechanisms of fatigue development can include altered electrolyte concentration, metabolite content, and calcium handling. Recently, posttranscriptional modifications of proteins associated with the contractile filaments have also proven important in regulating cardiac muscle function [14], [15]. Such modifications have been recently linked to heart failure [16]. How these protein modifications affect skeletal muscle function is less well known, but some reports have suggested that posttranslational modification of MLC2 is important in regulating skeletal muscle contraction [14], [17].

No experiments so far have been designed to evaluate how muscle function from animals with post infarction heart failure might change during shortening, isotonic contractions. Contrary to the hypothesis that shortening contractions would reveal an even more attenuated muscle function than isometric contractions, we found that skeletal muscle from heart failure rats tolerated shortening contractions very well, even better that the sham animals. MLC2 dephosphorylation could play an important role in fatigue development.

## Methods

All animal experiments were conducted in accordance with current regulations and approved by the Norwegian Animal Research Authority, approval ID 2310. Adult male Wistar rats (Wistar Hannover, Taconic, Skensved, Denmark) were kept in a temperature-, humidity- and light-controlled (12∶12 hour light-dark cycle) environment. Animals had access to standard rat chow (B & K Universal, Oslo, Norway) and water ad libidum, and were caged for ≥1 week after arrival from the supplier prior to inclusion in the study. A total of 98 animals were used.

### Induction of congestive heart failure

Rats were anesthetized with 1:3 O_2_-N_2_O with ∼2% isoflurane (Forene® Abbott no. 506949), intubated, ventilated, and subjected to ligation of the left coronary artery as described by Tønnessen *et al* [18]. Sham-operated animals, where the coronary artery was exposed but not ligated, served as controls (sham). Postoperatively, the rats were administered 0.2 mg·kg^−1^ buprenorphine (0.3 mg/ml) and kept under daily surveillance. With signs of severe discomfort animals were anaesthetized and sacrificed by neck dislocation.

Six weeks after induction of myocardial infarction, rats were once again anesthetized, intubated and placed on a heated (37°C) operating table. The right carotid artery was localized and a pressure sensitive catheter (SPR-407, Millar Instruments, Houston, TX, USA) was inserted and maneuvered retrogradely through the artery into the left ventricular cavity. Based on the conclusions from Sjaastad *et al* [19], only post infarction rats with left ventricle end diastolic pressure (EDP) >15 mmHg were included in the CHF group. In a subset consisting of 8 sham and 8 chf-animals, echocardiographic examinations were performed.

### Surgical preparation

The right leg was skinned from the knee down. The Achilles tendon was cut and the soleus muscle (SOL) was dissected free from surrounding tissue except for the proximal connection to tibia and fibula. The blood supply was left intact. A combined force and length transducer (model 305B, Aurora Scientific, Ontario, Canada) was connected to the distal tendon and platinum electrodes were positioned at the proximal and distal end of the muscle. The ankle and the middle region of the tibia were clamped, leaving the knee immobile and stable. The muscle core temperature was maintained at 37°C by continuous flow of heated 0.9% NaCl across the epimysium of the muscle. During experiments, the pressure-sensitive catheter was positioned in the carotid artery, making it feasible to continuously measure blood pressure during the experiments. Because the anesthetics employed can be cardiodepressive [20], special attention was paid to any change in systolic blood pressure. At the end of the experiment, animals were killed by neck dislocationwhile still anaesthetized.

### Stimulation protocol

The SOL was stimulated (Pulsar 6bp, FHC Brunswick, ME, USA) supramaximally (8 V) and muscle length was adjusted to obtain maximum isometric force at 1Hz and 100 Hz (F_max_). Increasing voltage did not increase force or shortening at any stage of the protocol and 100 Hz stimulation was tested to induce F_max_. During the entire stimulation protocol, the SOL was load-clamped at a load representing one third of F_max_ (afterload) for that individual muscle; hence the muscle shortened after reaching that force during stimulation. At this load, muscles are able to produce maximum power, and fatigue most rapidly [8]. The muscle was stimulated intermittently (1 s on and 1 s off) at 30 Hz for 100 s or 15 min, with 1 ms pulse duration.

The SOL of Wistar rats consists of 94% slow twitch fibers [21], and it has been shown that 30 Hz is close to the physiological firing rate in SOL during activity [22]. Also, by stimulating intermittently perfusion is likely maintained in the same way as rotation between motor units can ensure perfusion *in vivo*. During the period (1 s) without electrical stimulation, the muscle was re-extended to its pre-shortening length by the force represented by the pre-clamped load, one third of F_max_. Force, muscle length, muscle surface temperature, aortic blood pressure and stimulation pulse were sampled at 2000 Hz throughout the protocol. At six specific time points (0 s, 100 s, 300 s, 450 s, 600 s and 900 s), the isometric relengthening curve was fitted to the biexponential decay equation, y = y_0_+a^(−x/b)^+c^(−x/d)^, where x is time in ms and y is tension in N. The time constants obtained from this fit (b and d) where recorded as tau1 and tau2, respectively. See Table 1 for definitions of parameters calculated from the force and length recordings.

**Table 1:** Definitions of parameters calculated from the force and length recordings.

T_bl_	Baseline tension
F_max_	Maximum isometric force
V_0_	Unloaded shortening velocity
Afterload	1/3 of F_max_
L_0_	Optimum resting muscle length
dF/dt_max_	Rate of isometric force development (30 and 100 Hz)
−dF/dt_max_	Isometric relaxation rate (rate of force decline)
dL/dt_max_	Isotonic shortening velocity
−dL/dt_max_	Isotonic relengthening velocity
S_max_	Maximum shortening
tau1	Time constant of the rapid component of force decline (the isometric relaxation rate)
tau2	Time constant of the slow component of force decline (the isometric relaxation rate)

### Unloaded shortening velocity, isometric maximum force and contraction rate

Maximal isometric force (F_max_) and rate of isometric force development (100 Hz) were obtained before the stimulation protocol and after 100 s and 15 min of stimulation. The muscle was prepared as described above, and stimulated at the clamped optimal length at 100 Hz. A “slack test” was simultaneously performed to determine V_0_: The time until the muscle regained pull on the force transducer after a sudden, variable shortening (2–6 mm) from F_max_ was plotted on the x-axis against the imposed shortening distance on the y-axis. V_0_ was defined as the slope of a linear fit to this plot. The rate of isometric force development (100 Hz) was determined by using TableCurve (TableCurve 2D v5.01, SYSTAT Software Inc., San Jose, CA, USA) to analyze the force recordings.

### Metabolites

Working muscles were harvested after 100 s or 15 min of stimulation and plunged into liquid nitrogen as quickly as possible, normally within 5 s after the last stimulation. The non-working SOL of the contralateral leg served as control. ATP and CrP were determined by HPLC [23] and lactate by a fluorometric enzymatic coupled assay [24].

### Myosin light chain (MLC) analysis

Myofibrillar proteins were separated by glycerol/SDS polyacrylamide gel electrophoresis. The gels were stained sequentially by ProQ Diamond and SYPRO Ruby (M33305, Invitrogen, Oslo, Norway) for phosphorylated and total protein, respectively. Bands were detected by a Typhoon laser scanner (9410, GE Healthcare, Oslo, Norway), and quantified by Imagequant (GE Healthcare, Oslo, Norway). Phosphorylation was quantified at 100 s and 15 min of isotonic stimulation by dividing the staining intensity reflecting phosphorylation level by the staining intensity of the MLC2s protein band. Values are presented as level of phosphorylation relative to the control muscle. MLC2s bands were identified by Western blotting (ALX-BC-1150-S, Clone F109.3E1, Alexis, AH Diagnostics, Oslo, Norway).

### Statistics

Values are average ± SEM. Differences between groups (except parameters derived from the isotonic stimulation protocol) were tested using one-way ANOVA supplemented with post hoc Newman-Keuls test. A p-value <0.05 was considered significant. The parameters derived from the isotonic stimulation protocol were analyzed using natural splines [25] with six degrees of freedom, obtained using the ns function in the statistical programming language R (R Development CoreTeam, 2008). Natural splines is a statistical technique for analyzing time course data and allows detection of local group differences at one or a few time points. The values were considered different when p was below 0.05. Statistical analyses were performed with SigmaPlot 11.0 (Systat Software) and R: A language and environment for statistical computing (http://www.R-project.org).

## Results

### Animal characteristics

In comparison with sham, chf animals exhibited elevated EDP, lower systolic blood pressure, and dilated left ventricular dimensions during diastole (163% compared to sham, Table 2). Fractional shortening (defined as maximal left ventricular luminal diameter in systole relative to the diastolic diameter) was reduced in chf to one fourth of the level in sham, demonstrating severely depressed systolic left ventricular function. chf rats also exhibited clinical signs of pulmonary congestion such as tachypnoea and deeper respiration. This was confirmed by a doubling of left atrial diameter and lung weight in chf compared to sham (Table 2).

**Table 2:** Characteristics of the experimental groups.

	SHAM	CHF
BW (g)	398±7 (42)	382±5 (33)
SystBP (mmHg)	124±2 (40)	108±2 (28)*
EDP (mmHg)	2±0.2 (41)	27±1.1 (32)*
LW (g)	1.5±0.02 (31)	3.6±0.12 (32)*
LAd (mm)	3.5±0.1 (8)	7.1±0.3 (8)*
LVDd (mm)	6.0±0.2 (8)	9.8±0.3 (8)*
LVDs (mm)	2.9±0.2 (8)	8.5±0.3 (8)*
FS (%)	51±2 (8)	13±1 (8)*

BW: body weight; LW: lung weight; LAd: left atrial diameter; LVDd: left ventricular diastolic diameter; LVDs: left ventricular systolic diameter; FS: fractional shortening.

All values are average ± SEM (n)

*p<0.05 vs SHAM.

### Isometric contractile properties of the SOL during the fatigue protocol

**Figure 1:**
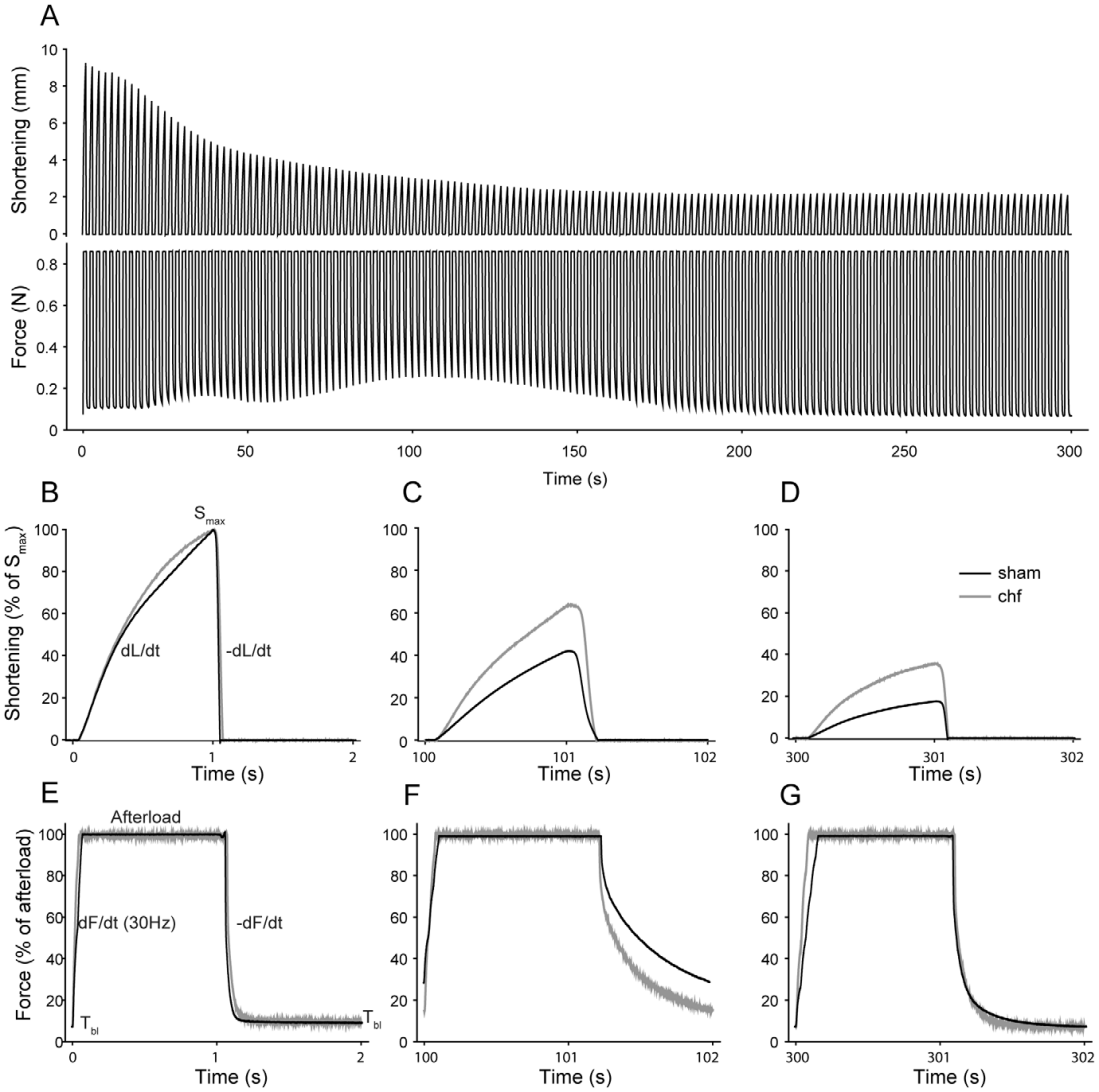
Dynamic exercise protocol. *A*, Representative shortening (upper) and force (lower) tracings of the first 5 min of isotonic exercise in SOL from a sham animal. During the complete cycle, the muscle is load-clamped at an afterload representing 1/3 of maximal force (F_max_). *B,* Expanded view of the shortening tracings from the first exercise cycle (1 s stimulation, 1 s rest). When afterload is attained, the muscle shortens till S_max_ and stimulation ends after which the muscle is passively relengthened by the afterload to resting length. E, Expanded view of the force tracings from the first exercise cycle (1 s stimulation, 1 s rest). As stimulation starts from resting tension (T_bl_) force rises and reaches the afterload. This force is maintained until stimulation ceases and the developed tension in the muscle decreases. *C* and *F*, shortening and force tracings after 100 s of exercise and *D* and *G*, tracings after 5 min of exercise. Representative sham and chf animal are shown in black and gray, respectively.

**Figure 2:**
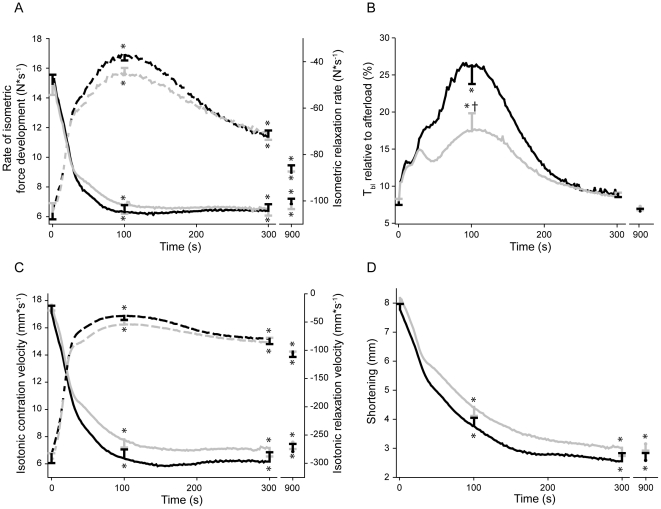
Isometric and isotonic properties during dynamic exercise. The rates were calculated for every stimulation cycle. The complete tracing is shown for the initial 5 min, while the subsequent 10 min is not shown. The rates at 15 min are indicated. Black and gray lines are responses from SOL of sham (n = 22) and chf (n = 24) rats, respectively. *A,* Rate of isometric force development during the exercise protocol (*solid lines:* dF/dt_max_ (confer Figure 1E)) and rate isometric force decline (*dashed lines:* −dF/dt_max_ (confer Figure 1E)). Note the transient nature of −dF/dt_max_ in contrast to dF/dt_max_. *B*, Baseline tension (T_bl,_ confer Figure 1E) before a new stimulation cycle starts. *C*, Isotonic shortening velocity (*solid lines:* dL/dt_max_ (confer Figure 1B)) and isotonic relengthening velocity (*dashed lines:* −dL/dt_max_ (confer Figure 1B)) during shortening exercise. Note that±dL/dt_max_ do not show the same transient behavior as −dF/dt_max_ in Panel A. *D*, Shortening, (S_max,_ confer Figure 1B). *A*–*D*, Bars at 0 s, 100 s, 300 s and 900 s time points are SEM. *p<0.05 vs. 0 s. †p<0.05 vs. sham.

In resting muscles there were no significant differences in SOL force-frequency relationships between sham and chf (data not shown). During the stimulation protocol (see Figure 1A for representative tracings), the maximal rate of isometric force development (dF/dt_max_) gradually decreased in both groups, without significant differences between them at any time point (Figure 2A). There was a rapid initial drop in dF/dt_max_ in the first 40 s followed by a modest reduction during the next 60 s (to 42% of the initial rate for sham and 46% for chf, p<0.001 vs initial rates for both groups). After 100 s of intermittent stimulation, there was no further change in dF/dt_max_, and after 15 min of stimulation dF/dt_max_ was 45% the initial value for sham and 46% of the initial rate for chf.

**Figure 3:**
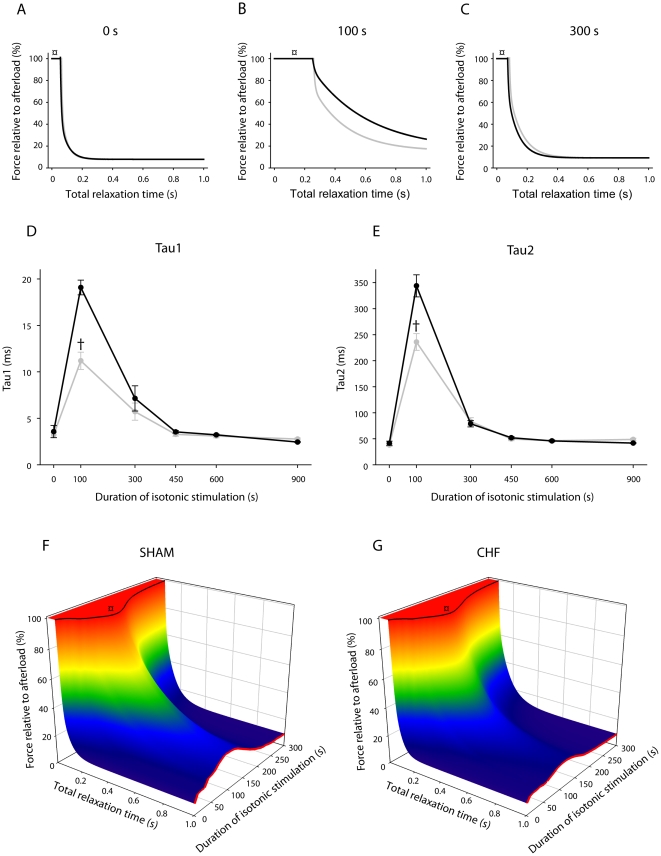
Isometric force decline. *A, B and C*: Representative tracings of the isometric relaxation curves after 0 s (*A*), 100 s (*B*) and 300 s (*C*) of the exercise protocol. Note the time lag before force decline starts (marked with ¤) due to time required for relengthening. The transient slowing of isotonic relengthening peaked after 100 s of stimulation (confer Figure 2C and 3F and G). Sham in black and chf in gray. *D and E*: Time constants (tau1 (*D*) and tau2 (E), average values for sham (n = 22) and chf (n = 24)) from the isometric force decline at 0, 100, 300, 450, 600 and 900 s of stimulation calculated from a fitted biexponential decay curve. Error bars are ± SEM. Sham in black and chf in gray. †p<0.05 vs. sham. *F* and *G:* Averaged force recordings (y-axis) during the period of relaxation (isotonic and isometric phases, x-axis), assembled for the first 5 min of exercise (z-axis) for sham (*F*, n = 22) and chf (*G*, n = 24). The time period for relengthening before force decline starts is indicated (black line marked with ¤, confer same symbol in panel *A*, *B* and *C*). T_bl_ is indicated with a red line. Note the transient rise after 100 s in both groups (confer Figure 2B and 3A, B and C). Since there is no difference between the two groups regarding the isotonic relengthening (black line, *F* and *G* (¤)), the attenuated increase in T_bl_ in chf compared to sham after 100 s (red line, *F* and *G*) is due to differences in the isometric force decline.

Throughout the stimulation protocol, rate of isometric force decline was maximal immediately following cessation of muscle relengthening (−dF/dt_max_). There was no difference between the groups, with an initial rapid decline in −dF/dt_max_ the first 40 s (Figure 2A) followed by a slower progression during the subsequent 60 s (to 36% and 43% the initial value for sham and chf, respectively. p<0.001 vs initial rate for both groups). Unlike dF/dt_max_, the −dF/dt_max_ was partially restored after 15 min to 85% and 81% of the initial value for sham and chf, respectively. However, the −dF/dt_max_ does not adequately describe the complete relaxation phase. Therefore, we fitted biexponential decay equations to isometric force recordings. The overall fit was excellent (r^2^>0.99) and the difference between sham and chf was striking (Figure 3D and E). The initial time constants were not different between the groups. The time constant for the first component (tau1) was initially 3.6 ms in sham and increased to 19.1 ms at 100 s. For the chf group tau1 increased from 3.2 to 11.2 ms, p<0.05 vs sham at 100 s. The time constant of the second slow component (tau2) also increased considerably. Initially, tau2 was 41.5 and 38.1 ms in sham and chf, respectively, and increased to 343.9 and 236.0 ms at 100 s (p<0.05), indicating more rapid isometric relaxation in chf. Both tau1 and 2 decreased in the course of the subsequent 200 s, to 7.1 and 5.7 ms (tau1) and 78.9 and 83.4 ms (tau2) for sham and chf, respectively. Both time constants stabilized at values close to initial values after 450 s of stimulation. The other parameters from the biexponential decay curve (y_0_, a and c) varied throughout the protocol without significant differences between the groups.

The baseline tension (T_bl_, Figure 2B) reflects the tension before the next stimulation and is dependent on the rate of force decline (−dF/dt) and available time for isometric relaxation. The isometric relaxation commences after the muscle is re-extended to its pre-shortening length (confer Figure 1). Since the isotonic relengthening velocity (−dL/dt) decreased during the initial 100 s the time available for isometric relaxation was reduced by 21% during the initial 100 s for sham and for chf. However, this was not enough to affect T_bl_. Thus, the most important determinant of T_bl_ was the two time constants of the isometric force decline (Figure 3). T_bl_ showed a three-bumped transient rise during the initial 5 min of the protocol before stabilizing at near-initial tension. The shape of this curve was similar for both groups, with T_bl_ “peaking” after approximately 8, 40 and 100 s (Figure 2B). T_bl_ increased significantly less in the chf group as compared to sham (from 8% of the afterload to 26% and 18% in sham and chf, respectively; p<0.001for sham vs chf and vs initial tension for both groups). After 100 s T_bl_ was gradually restored in accordance with the decrease of tau1 and tau2 of the isometric relaxation and, after 160 s of the fatigue protocol, there were no significant differences between the groups. T_bl_ stabilized after 300 s at 9% of the afterload in both groups.

### Shortening properties of the SOL during the fatigue protocol

The maximal isotonic shortening velocity (dL/dt_max_) declined during the protocol, with a rapid decline during the initial 40 s (Figure 2C). The next 60 s was followed by a more moderate decrease (to 37% of the initial velocity for sham and 44% for chf, p<0.001 vs initial velocity in both groups). After 100 s, dL/dt_max_ remained stable, and after 15 min the velocity was 39% and 43% of the initial velocity for sham and chf, respectively. There were no significant differences between the groups at any time point.

The isotonic relengthening velocity (−dL/dt_max_) also decreased rapidly during the initial 40 s of the protocol, and then declined less markedly in the next 60 s (at 100 s, −dL/dt_max_ = 14% (sham) and 18% (chf) of the initial velocity, p<0.001). After this time point −dL/dt_max_ increased modestly. After 5 min the rate was 28% (both groups), and after 15 min the −dL/dt_max_ was 35% (sham) and 37% (chf) of the initial velocity. There were no significant differences between the two groups.

During the first 40 s, muscle shortening (S_max_) dropped rapidly in both groups (to 67% and 72% the initial length for sham and chf, respectively (p<0.001); Figure 2D). The decline of S_max_ was nearly mono-exponential during the protocol, and flattened out after 5 min at 33% (sham) and 37% (chf) of the initial length. At the end of the protocol, shortening was 32% and 39% of the initial S_max_. Although not significantly different at any time point, SOL from chf animals tended to shorten more than sham throughout the entire stimulation period. During the initial 300 s accumulated shortening (proportional to total work) of SOL in sham animals was 555±35 mm compared to 633±38 mm in SOL from chf (p = 0.14).

### Slack test and isometric properties under tetanic stimulation

F_max_, V_0_ and dF/dt (100Hz) were measured before and after 100 s and 15 min of isotonic stimulation. Initially there were no differences between the groups in any of these parameters (Table 3). All parameters dropped significantly by a maximum of roughly 25% during the first 100 s of the protocol. Following 15 min of isotonic stimulation, F_max_ and dF/dt_max_ tended to be restored in sham compared to the level at 100 s whereas V_0_ was not restored. In chf, F_max_ was further reduced, whereas V_0_ and dF/dt (100 Hz) increased compared to the level at 100 s.

**Table 3:** Isometric properties of SOL from CHF and SHAM rats stimulated at 100 Hz at rest and after 100 s and 15 min of the isotonic contractions.

		F_max_ (N)	V_0_ (mm·s^−1^)	dF/dt (100 Hz) (N·s^−1^)
	CTR	2.4±0.05 (46)	174±5 (9)	39.4±3.1 (8)
SHAM	EX – 100 s	1.9±0.09 (12)*	160±4 (12)*	29.7±2.4 (12)*
	EX – 15 min	2.1±0.07 (9)*	155±5 (8)*	34.5±1.3 (9)
	CTR	2.3±0.04 (32)	183±3 (7)	42.2±2.0 (9)
CHF	EX – 100 s	2.0±0.10 (7)*	150±6 (7)*	26.7±2.2 (6)*
	EX – 15 min	1.9±0.11 (8)*	158±7 (8)*	28.8±2.6 (5)*

CTR – contralateral, resting SOL. EX – exercising SOL.

All values are average ± SEM (n)

*p<0.05 vs CTR.

### Metabolites

Table 4 shows that during the first 100 s of stimulation there was a significant drop in CrP and ATP levels in working SOL from both sham and chf. Also, there was a significant three- to fourfold increase in lactate level in exercising muscles, which was not significantly different between chf and sham. The metabolite concentrations at 100 s of isotonic stimulation remained steady through 15 min, with the exception of a significant decrease in lactate concentration in chf. Phosphate (P_i_) was not measured, but judging from the fall in CrP and ATP, P_i_ levels probably increased to the same extent in the two groups. Levels of ATP, CrP and lactate were not significantly changed in contralateral resting muscles harvested after 100 s and 15 min of the protocol.

**Table 4:** Metabolites in resting SOL and SOL from CHF and SHAM rats after 100 s and 15 min of isotonic contractions.

		ATP	CrP	Lactate
	CTR	5.0±0.2 (18)	15.7±0.8 (18)	3.3±0.5 (14)
SHAM	EX – 100 s	2.5±0.4 (10)*	3.3±1.0 (10)*	10.3±1.9 (8)*
	EX – 15 min	1.9±0.6 (7)*	2.6±0.5 (7)*	9.3±1.3 (6)*
	CTR	5.4±0.2 (32)	15.2±0.9 (32)	2.8±0.3 (29)
CHF	EX – 100 s	2.3±0.3 (19)*	4.0±0.5 (19)*	11.6±1.2 (19)*
	EX – 15 min	2.2±0.4 (16) *	5.0±1.3 (16)*	4.6±0.9 (13)* † ¤

CTR – contralateral, resting SOL. EX – exercising SOL.

All values are in mmol·(kg wet weight)^−1^, average ± SEM (n).

*p<0.05 vs CTR,

† p<0.05 vs EX – 100 s,

¤ p<0.05 vs SHAM EX – 15 min.

### MLC phosphorylation

**Figure 4:**
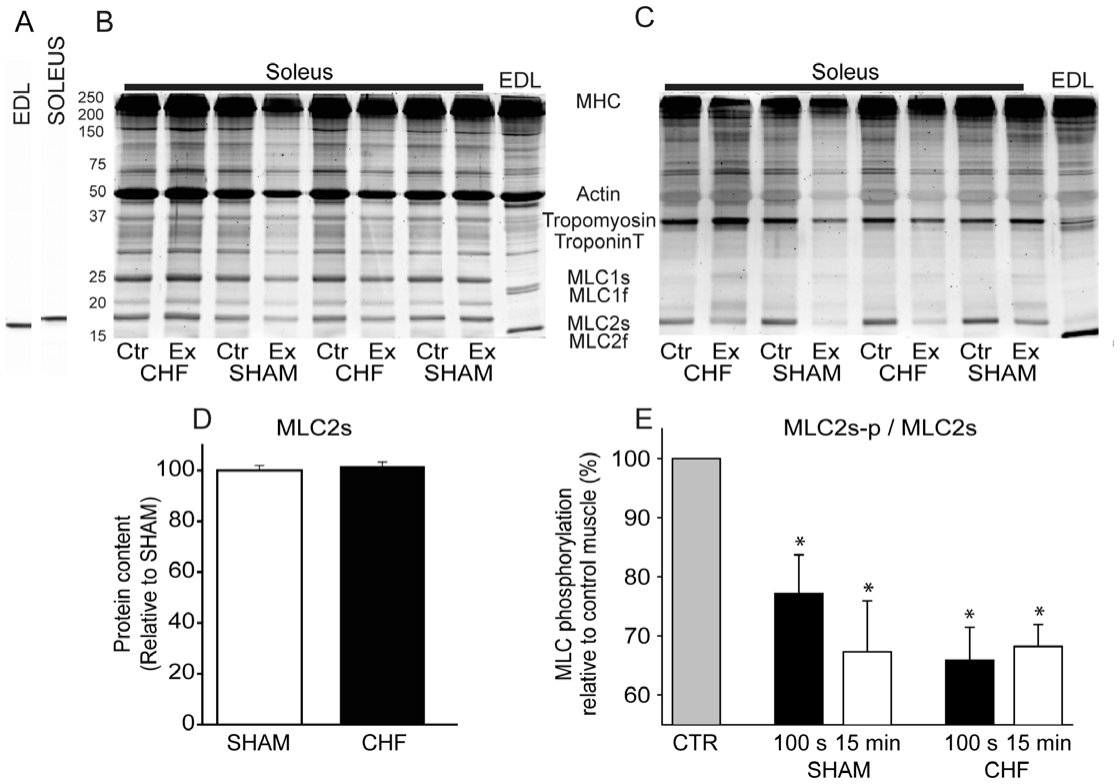
MLC phosphorylation. *A,* Representative immunoblot of extensor digitorum longus (EDL) and Soleus probed with monoclonal MLC2 antibody. *B*, Total protein, stained with SYPRO Ruby for total protein content. *C*, Same gel as in panel B stained with Pro Q Diamond for phosphorylated proteins. *D*, Spot density in MLC2s in sham and chf animals related to spot density in sham animals. *E*, MLC2 phosphorylation in resting (CTR) and exercising SOL normalized to the resting control muscle. Two time points during exercise (100 s and 15 min) were investigated. Average data, sham (100 s and 15 min, n = 5) and chf (100 s and 15 min, n = 15). Bars are + SEM. *p<0.05 vs. CTR.

After 100 s of the protocol, MLC2s was significantly dephosphorylated in the exercised SOL compared to the control leg, by 23% and 34% for sham and chf, respectively (Figure 4). After 15 min MLC phosphorylation was reduced by 33% and 32% of control leg values in the sham and chf group, respectively. Thus, there was no significant change in phosphorylation level compared to the 100 s time point for either group, and no significant differences between groups at any time point.

## Discussion

This is the first experimental study to examine the *in situ* shortening performance of SOL of animals with chf at physiological temperatures. Previous animal studies have focused solely on fatigue development during isometric contractions and we have previously reported reduced fatigue resistance in SOL from chf rats using such a protocol [4]. However, fatigue is typically task dependent, so important aspects of the development of fatigue that occur during shortening contractions may have escaped detection. Surprisingly, we did not observe any significant difference of initial performance for any contractility parameter between the sham and the chf group (Table 5). The main finding in this study was that during a fatiguing exercise protocol, the time constants describing the isometric relaxation phase (force decline after the muscle was relengthened) increased significantly less in chf animals compared to sham, resulting in an attenuated increase in T_bl_ in chf at 100 s compared to sham. Further, during the exercise protocol the changes in F_max_ and V_0_ were remarkably similar between the two groups, as were the alterations in ATP and CrP. Hence, performance of the soleus muscle in chf rats was in fact superior to that of sham animals.

**Table 5:** Contractile properties of SOL from CHF and SHAM rats during the first stimulation cycle of the dynamic fatigue protocol.

	SHAM	CHF
Animals	22	24
dF/dt_max_ (N·s^−1^)	15.0±0.6	14.8±0.6
−dF/dt_max_ (N·s^−1^)	−104.4±3.6	−102.2±4.8
dL/dt_max_ (mm·s^−1^)	17.1±0.5	17.8±0.4
−dL/dt_max_ (mm·s^−1^)	−283.9±15.6	−298.2±12.0
S_max_ (mm)	7.8±0.2	8.2±0.2
T_bl_ (% of afterload)	7.9±0.3	8.1±0.4
Tau1 (ms)	3.6±0.6	3.2±0.3
Tau2 (ms)	41.5±4.0	38.1±2.8

See Figure 1B and E. All values are average ± SEM.

The muscle's internal energy demand is at least 3 times larger during shortening (working) than during static (non-working) contractions, and our protocol was designed to achieve maximum power and hence maximum energy demand [6], [26]. The changes in ATP, CrP and lactate during the first 100 s of the exercise protocol were notably similar in the two groups, contradicting the finding that muscle from chf rats has a reduced glycolytic capacity [27]. Subsequently, muscle metabolite levels were maintained during further exercise, with the exception of lactate in the chf group which fell considerably. This finding strongly indicates that aerobic metabolism and ATP supply to the SOL were not impaired in chf. Thus, it can be speculated that reported changes in mitochondrial function and metabolism in skeletal muscle from experimental animals and patients with heart failure [7] are not of sufficient severity to compromise energy delivery to ATP requiring processes during exercise. In fact in the SOL of chf animals lactate may have been oxidized during continued exercise. This agrees with studies that have reported a lower increase in muscle lactate in patients with heart failure compared to controls [28], [29]. Maintained aerobic capacity in skeletal muscle is also in line with recent findings from an exercise study in heart failure patients from our laboratory [30].

The isometric force decline is particularly sensitive to exercise, and a decreased rate of force decline is a characteristic sign of a fatigued muscle [31]. Therefore, it is interesting that during shortening contractions, the largest difference between sham and chf animals was observed during the isometric phase. Also of note is that in rats the slowing of force decline is a transient phenomenon, as we have previously reported using the same protocol with isotonic or isometric contractions [32]–[34]. Interestingly, in a human exercise model where isometric contractions at various intensities were carried out by the vastus lateralis muscle, decline of force became faster during exercise in line with the restitution we presently observed from the 100 s time point [35]. We can conclude that the increased energy demand introduced with shortening contractions did not aggravate the initial slowing or the subsequent acceleration of force decline. The cause of slowing could involve alterations in metabolites or signals that are transiently altered but show restitution during continued exercise. In normal rats partial restitution of CrP and lactate was observed during the exercise protocol [14]. In the sham group no such associations could be identified. In the chf group muscle lactate was transiently elevated, but on the other hand the slowing of force decline was much less pronounced than in sham. Taken together these observations indicate that no single factor can explain the transient slowing of force decline.

The rate of decline of force is partly dependent on cross-bridge kinetics and partly on the rate of decline of Ca^2+^ in the cytosol [31]. Rate of Ca^2+^ reuptake into the SR is not regarded to be of great importance for velocities and rates of relaxation except for the slower phase of force decline (tau2) [36]. Thus the major focus in the literature has been on changes in metabolites and their effects on cross-bridge kinetics. In line with the literature, we did not observe differences between groups in the resting concentration of ATP and CrP in SOL [37]. Slower relaxation rate is reported to be correlated to reduced levels of the high energy phosphates, and experiments indicate that increased ADP due to breakdown of ATP (Table 4) could delay both early (tau1) and late (tau2) force decline kinetics [38], [39]. Also, decrease in CrP with concomitant rise of P_i_ can slow relaxation [40]. Finally, lactate accumulation has been reported to reduce the myosin-actin detachment rate, thereby reducing the rate of force decline [41]. Thus, the initial slowing of force decline can very likely be related to one or several of the observed changes in metabolites. However, why is slowing of force decline transient and why is it less pronounced in the chf group when metabolites are not different?

Before returning to the question of why rate of force decline shows restitution, a few comments on the results of the slack test are required. F_max_ and dF/dt (100 Hz) were moderately reduced at 100s in line with what is commonly reported with fatigue [42]. This could result from accumulation of P_i_ due to the breakdown of CrP. It has been shown that high P_i_ will cause a rightward shift of the force-pCa curve [43]. The slightly depressed V_0_ was probably related to elevated levels of ADP [38], [39]. Lactacidosis and H^+^ ions can depress both F_max_ and V_0_ [44], [45], even at physiological temperatures [46]. Despite a partial restitution of the lactate concentration in SOL from chf-animals there was no restoration of either F_max_ or V_0_. This indicates that lactate is not a major contributor to the sustained reduction in these parameters following 15 min of stimulation, and supports the notion that cytosolic lactate accumulation might not be a major factor in the development of fatigue [47]. However, lower peak cytosolic [Ca^2+^] is likely related to the small reduction in F_max_ [44], [48] and a concomitant slower decline of the Ca^2+^ transient could have several explanations. First, this could be due to the inhibitory effect of elevated P_i_ on the SERCA pump in combination with acidosis after 100 s [49]. However, the rate of decline of cytosolic Ca^2+^ is also influenced by the size of Ca^2+^ leak from the SR through the ryanodine receptor. In fact a Ca^2+^ leak via the SERCA pump has also been reported under these conditions [50]. An increased leak of Ca^2+^ from the SR will counteract the pumping of Ca^2+^ by SERCA into the SR and slow the rate of decline of cytosolic Ca^2+^ in SOL during activity. Although this SERCA leak appears to be most prominent in fast twitch fibers, the mechanism could contribute to the increase in tau2 after 100 s. The amount of SERCA of slow twitch muscle has been reported to be upregulated in experimental models of heart failure [51], which could at least partly explain the differences between sham and chf in relaxation rate and tau2 after 100 s. Interestingly, we have shown that Ca^2+^ leak from the SR in biopsies from the vastus lateralis muscle is actually lower in chf patients than in control subjects which could fit with a less pronounced slowing of force decline in the SOL of chf rats [52]. One could speculate that a decline in the Ca^2+^ leak from the SR would cause a more rapid decline of the Ca^2+^ transient and that this could explain why rate of decline of force was gradually restored during continued exercise after 100 s. The proposal that the balance between Ca^2+^ leak and reuptake determines the rate of isometric relaxation (the slow component, tau2) needs further investigation.

MLC2 phosphorylation is commonly reported to be increased during exercise and is linked to the post tetanic twitch potentiation in fast twitch skeletal muscle [17]. We were therefore surprised to find a reduction in MLC2s phosphorylation already after 100 s of shortening contractions in both groups. This suggests a physiological role for altered MLC2 phosphorylation level also in slow twitch muscle. However, most analysis of MLC2 phosphorylation has been undertaken in fast twitch muscle fibers contracting isometrically where phosphorylation seems to lower contraction velocity in concert with low pH and high P_i_ [53]. During shortening contractions it seems that MLC2s is regulated differently since it was dephosphorylated [14]. Also, the enzyme systems engaged in MLC2 phosphorylation and dephosphorylation show fiber type specific differences [14], [54], [55], and thus could partly explain why our results in slow muscle fibers differ from what is reported in fast muscle fibers. MLC2s phosphorylation moves the myosin head closer to actin [56]. If dephosphorylation does the opposite, the likelihood of myosin and actin interaction during stimulation will be reduced. This could explain the parallel reduction of the rate of isometric force development at 30 Hz, S_max_, and the velocity of isotonic shortening during the stimulation protocol. There were no statistical differences in the phosphorylation level in exercised SOL for the groups, which fits with the lack of significant differences for these three parameters.

Several publications have shown decreased fatigue resistance to isometric contractions in skeletal muscle of heart failure animals [4], [51]. The present study is the first to include results from shortening *in situ* contractions in chf rats at physiological temperatures. In conclusion, the performance of SOL from rats with chf is remarkably similar to sham. The slowing of isometric relaxation was the only mechanical parameter which differed between the two groups, with an attenuated reduction in the failing group. It is possible that different fatigue mechanisms are at work depending on mode of contraction. We speculate that patients with chf may tolerate cycling on an ergometer bike (isotonic, shortening exercise) better than climbing stairs, which to a greater extent is dependent on isometric force development. The functional importance of a maintained isometric relaxation rate during exercise is probably even underestimated in the present study, as the duty cycle employed here (0.5 Hz) is lower than limb frequency during human running (2 Hz) [57]. These results could have important implications for the evaluation of skeletal muscle function and the design of training programs for the heart failure patient.
